# Family meals are associated with lower substance use in female adolescents

**DOI:** 10.1111/famp.13039

**Published:** 2024-07-31

**Authors:** Danny Rahal, Michael R. Irwin, Andrew J. Fuligni

**Affiliations:** ^1^ Psychology Department University of California, Santa Cruz Santa Cruz California USA; ^2^ Cousins Center for Psychoneuroimmunology University of California, Los Angeles Los Angeles California USA; ^3^ Department of Psychiatry and Biobehavioral Sciences University of California, Los Angeles Los Angeles California USA; ^4^ Department of Psychology University of California, Los Angeles Los Angeles California USA

**Keywords:** adolescent, family dinner, family meals, substance use

## Abstract

Adolescents, especially female youth, who have more family meals tend to be at lower risk for substance use. The present study tested whether family meals relate to substance use count and frequency during high school, whether associations differ by gender, and whether other family‐related variables explain these associations. A community sample of 316 adolescents (*M*_age_ = 16.40, *SD* = 0.74; 56.96% female; 41.77% Latine, 23.10% Asian American, 29.11% European American, 6.01% from other ethnic backgrounds including Middle Eastern and African American) reported the number of substances they have ever used and how often they used alcohol, marijuana, and cigarettes, and completed measures of parental support and family cohesion. Across 15 days, they reported whether they had a family meal, got along with parents, and spent leisure time with their family each day. Regression models tested associations between frequency of family meals and substance use, whether associations differed by gender, and whether associations were explained by other family‐related variables. Results indicated that more frequent family meals were associated with lower substance use count and less frequent alcohol, marijuana, and cigarette use among female adolescents but not male adolescents. Other daily family experiences were unrelated to substance use, and family meal frequency was independently related to lower substance use after accounting for parental support and family cohesion. Taken together, more frequent family meals in high school may reduce substance use risk for female adolescents, and interventions could consider promoting family meals in addition to other positive family values.

During adolescence, youth are at heightened risk of substance use initiation and escalation (Johnston et al., 2023). More frequent substance use in adolescence typically leads to greater substance abuse and dependence in adulthood (Grant & Dawson, 1997). Family Systems Theory and developmental risk models posit that family relationships and experiences including family rituals impact youth's behaviors and adjustment (Kerr & Bowen, 1988). Frequent family meals—or having a meal with another family member, including a sibling or caregiver—can be a stable routine that promotes family relationships and communication, with evidence suggesting stronger associations in female relative to male youth (Eisenberg et al., 2008; Fisher et al., 2007; White & Halliwell, 2011). However, few studies have tested whether adolescents who have frequent family meals may have lower substance use risk and whether other family activities and aspects of relationships (e.g., family cohesion and leisure time) may explain this association. In the present study, we examined whether family meals were related to adolescents' substance use count and frequency of alcohol, marijuana, and cigarette use, whether associations differed by gender, and whether associations were explained by other family activities and relationships.

Family meals are related to better adolescent well‐being and healthier dietary habits (Hammons & Fiese, 2011; Utter et al., 2017), but associations between family meals and substance use risk are relatively less consistent (Goldfarb et al., 2015). More frequent family meals have been related to lower risk for substance use, including lower frequency of alcohol, marijuana, and tobacco use both concurrently and years later (Eisenberg et al., 2004, 2008; Fulkerson et al., 2009; Levin et al., 2012; Sen, 2010), with stronger associations in female than in male adolescents (Eisenberg et al., 2008; Fisher et al., 2007; White & Halliwell, 2011). Family meals can serve as a family ritual, imparting stability within the home (Skeer et al., 2018). Youth who engage in these family rituals and routines tend to feel connected to their families, which may deter them from engaging in risky behaviors outside the home such as engagement with deviant peers or substance use (Brown et al., 2019; Fiese et al., 2002; Fulkerson et al., 2006). Routines such as family meals also have symbolic significance by fostering a shared family identity (Fiese, 1992; Wolin & Bennett, 1984). Attending family rituals including a regular dinnertime has been found to reduce at‐risk children's likelihood of alcoholism, and the disruption of such rituals is thought to contribute to the intergenerational transmission of alcoholism from parents to youth (Bennett et al., 1987; Wolin et al., 1980). Satisfaction with family rituals has been related to greater self‐reliance and other aspects of psychosocial development in late adolescence (Eaker & Walters, 2002).

These experiences may be particularly impactful for female adolescents because girls tend to be more affiliative than boys during adolescence (Cyranowski et al., 2000; Feingold, 1994). Evidence suggests that family interactions and relationships are more related to female adolescents' mental health and substance use compared to that of male adolescents (Dakof, 2000; Davies & Lindsay, 2004; Keijsers et al., 2010; Skeer et al., 2011; Telzer & Fuligni, 2013). For example, female adolescents tend to be more emotionally affected by interpersonal conflict (Chung et al., 2009; Flook, 2012) and physiologically linked with their parents during family conflict relative to male adolescents (Saxbe et al., 2014), both of which can contribute to psychopathology and risk‐taking behavior (e.g., Piehler & Dishion, 2014; Rahal, Alkon, et al., 2023; Rahal, Shirtcliff, et al., 2023).

Family meals also provide time for families to discuss daily events (Hammons & Fiese, 2011; Skeer et al., 2018). Adolescents report spending nearly half of the mealtime talking to their parents (Offer, 2013). Conversations can address specific topics, such as problems youth experience, or be more subtle and promote communication and convey concern (Fiese et al., 2006). More frequent family interactions allow parents to better detect changes in their adolescents' behaviors, such as emergence of risk‐taking behaviors or engagement with deviant peers (Hindelang et al., 2001; Perasso et al., 2021). Therefore, parents may identify and address changes in their children's behavior before they escalate, or children may be more hesitant or less motivated to escalate behavior if they believe their parents will become aware and involved. Indeed, more frequent family meals have been related to lower alcohol use through perceived parental involvement (Perasso et al., 2021). Female adolescents are more likely to report using substances to reduce distress, whereas male adolescents are more likely to report using substances to enhance positive arousal (Kuntsche et al., 2015; Pompili & Laghi, 2019). Family meals could be an outlet for discussion and buffer negative emotions; family meals have been found to reduce day‐to‐day emotional reactivity, engagement in avoidance‐oriented emotion regulation, and difficulties with emotion regulation (Armstrong‐Carter & Telzer, 2020; Hansson et al., 2017; Romano et al., 2022). By addressing stress and promoting well‐being, family meals may mitigate motivations for substance use in female but not male adolescents.

In spite of these posited mechanisms, prior research has been limited in its ability to assess how family meals may uniquely relate to substance use in adolescence. Prior studies suggest that parent–child relationships may partially explain how family meal frequency relates to well‐being (Goldfarb et al., 2015; White & Halliwell, 2010). Family meals may be related to lower substance use for adolescents by promoting parent–child relationships and family cohesion (i.e., family emotional closeness), as children with stronger family relationships may either avoid risk‐taking behaviors or discuss such behaviors with their parents. However, other studies suggest that family meals are related to substance use independently from familial closeness (Eisenberg et al., 2008; Fulkerson et al., 2006). Further, few studies have assessed whether other daily family experiences are similarly related to adolescent substance use. Family meals may be distinct from other family events that may be infrequent, less conducive to discussion, or prone to distraction (e.g., vacation, commutes to school, watching television). Taken together, research has been mixed regarding whether family meals uniquely relate to adolescents' substance use, in part due to methodological limitations including limited assessment of other daily family activities. Research is needed to determine whether family meals relate to lower substance use by cultivating parent–child relationships and family cohesion and whether other daily family experiences are similarly related to lower substance use in order to inform interventions.

The present study examined whether family meal frequency was related to substance use and whether associations differed by gender in high school students, given that substance use tends to escalate during this time (Johnston et al., 2023). Analyses were tested in a community sample of socioeconomically diverse youth because evidence suggests that low socioeconomic status may impose barriers to regular family meals (e.g., work conflicts, food insecurity; Middleton et al., 2020). In line with previous research (Eisenberg et al., 2004), adolescents who had more frequent family meals were hypothesized to have a lower substance use count and to use alcohol, marijuana, and cigarettes less frequently, especially for female adolescents due to aspects of socialization rather than biological differences from male adolescents. Whereas previous studies have asked participants to estimate their general frequency of family meals, which may bias reporting, participants in the present study completed daily checklists for 2 weeks, for which they reported daily family meals. To better understand how family meals may relate to substance use, we tested whether associations were maintained over and above other daily family experiences, specifically getting along with parents and spending leisure time with family, and were explained by parental support and family cohesion.

## METHODS

### Participants and procedures

A community sample of 316 adolescents (*M*_age_ = 16.40, SD = 0.74; 56.96% female; 41.77% Latine, 23.10% Asian American, 29.11% European American, 6.01% from other ethnic backgrounds including Middle Eastern and African American) from the 10th and 11th grades were recruited from four public high schools in Los Angeles County via in‐class presentations, mailings, and flyers between October 2011 and June 2012. Most participants resided in two‐parent households (68.7%) with an average family size of four members (M = 4.25, SD = 1.28).

Adolescents completed a psychosocial survey using an iPad or laptop in which they reported the number of substances they had ever used and their frequency of substance use, and a primary caregiver reported demographic information including family annual income, the highest level of education attained by each parent, and their child's gender (male or female), which always aligned with participant's self‐reported gender. Although designed in line with many national surveys during the period of data collection (Westbrook & Saperstein, 2015), gender responses were flawed by including terms referring to biological sex rather than gender (e.g., boy/girl or masculine/feminine) and not allowing open‐ended responses. Adolescents also completed a 2‐week daily checklist protocol in which they reported each night whether they experienced various events with family. On‐time completion for the 15 days was high; participants marked the exact date and time when the checklist was complete using an electronic stamper, and over 98% of checklists were completed before noon the next day. Daily methodologies are thought to indicate a person's general day‐to‐day patterns of behavior (Bolger et al., 2003). Sampling participants over 2 weeks can provide a snapshot of one's general family routines over the year, although scheduling conflicts and other barriers could emerge beyond the sampling period. Adolescents received $50 for completing the survey and two movie tickets for on‐time completion of checklists. Adolescents provided assent, and primary caregivers provided consent. All procedures were approved by the University of California, Los Angeles Institutional Review Board.

### Measures

Full measures are provided as supplemental information (see Appendix S2).

#### Substance use

As part of the psychosocial survey, participants retrospectively reported whether they had ever used any of the following substances: alcohol, marijuana, cigarettes, cocaine, crystal meth, illegal drugs, or any prescription drugs without a valid prescription. A sum substance use count was calculated for each participant, with a possible range from 0 (*never used any substances*) to 7 (*used all 7 substances in their lifetime*). If participants had used alcohol, marijuana, and cigarettes, they then reported frequency of use. For alcohol, participants reported how many days they had at least one drink of alcohol over the past year on a 10‐point scale (1 = *Never in the past year*, 2 = *1–2 days in the past year*, 3 = *3–11 days in the past year*, 4 = *one day a month*, 5 = *2–3 days a month*, 6 = *one day a week*, 7 = *two days a week*, 8 = *3–4 days a week*, 9 = *5–6 days a week*, 10 = *Every day*). For marijuana, participants reported how many days they used marijuana over the past year on the same 10‐point scale. Finally, for cigarettes, participants reported how many days they smoked cigarettes over the past 30 days on a 7‐point scale (1 = *Never in the past 30 days*, 2 *= 1–2 days*, 3 *= 3–5 days*, 4 *= 6–9 days*, 5 *= 10–19 days*, 6 *= 20–29 days*, 7 *= Every day*). For each frequency scale, participants who had never used the substance were coded as 0. All items were taken from the Youth Risk Behavior Surveillance System (YRBSS). Substance use was not measured daily because use of a daily protocol to assess infrequent events could introduce additional biases and underestimate adolescents' use, particularly for youth who only use occasionally or during special occasions (e.g., birthdays, when family is away). National data suggest that over half of 10th and 11th graders have never used alcohol or cannabis (Johnston et al., 2023), and rates were overall low for the present sample as expected.

#### Family meal frequency

Each day, adolescents reported on the checklist whether they ate a meal with their family (0 = *no*, 1 = *yes*), in line with prior checklist assessments of daily positive family interactions (e.g., Telzer & Fuligni, 2013). A sum was calculated across days, with 0 indicating that youth never had a family meal and 15 indicating that they had a family meal every day of the 2‐week checklist protocol. We used this broad item because the structure and timing of family meals vary across different households (Berge et al., 2019; Larson et al., 2013) and past studies differ in whether they define a meal by the number of people present and the time of the meal (Offer, 2013). A prior study using this item found that adolescents report better emotional well‐being and role fulfillment on days when they have a family meal, as well as reduced emotional reactivity to family conflict (Armstrong‐Carter & Telzer, 2020). This daily approach was used to rule out potential retrospective bias, and estimates are thought to indicate adolescents' general predisposition for having family meals in their daily lives.

#### Parental support

Participants reported parental support as part of the survey. Participants completed the parental support subscale of the Inventory of Parent and Peer Attachment (Armsden & Greenberg, 1987). They rated nine items regarding how close and supported by their parents they felt over the past month using a 5‐point scale (1 *= Almost Never*, 2 *= Once in a While*, 3 *= Sometimes*, 4 *= Frequently*, 5 *= Almost Alway*s; e.g., “I trusted my parents,” “I could count on my parents when I needed to talk.”). This scale has been well validated (Andretta et al., 2017; Gullone & Robinson, 2005; Wilkinson & Goh, 2014), and higher scores have been related to family interactions and higher adolescent well‐being (Gonzales et al., 2006; Yau et al., 2009). Items showed high reliability (*α* = 0.93), and an average was calculated across items.

#### Family cohesion

Participants also reported family cohesion as part of the survey using the Family Adaptability and Cohesion Evaluation Scale (FACES II; Olson et al., 1983). They rated 10 items, four of which were negatively worded, regarding their family's closeness and how often their family spends time together using the same 5‐point scale (e.g., “My family and I feel very close to each other,” “My family and I do things together”). This scale has also been well validated and widely used (Kouneski, 2000), and higher levels of family cohesion on this scale have been related to fewer depressive symptoms and higher self‐esteem later in life (Lin & Yi, 2019). Items showed high reliability (*α* = 0.87), and an average was calculated across items.

#### Frequency of daily family experiences

Using the daily checklists, participants reported whether they got along with their parents and whether they spent leisure time with their family each day (0 = *no*, 1 = *yes*). Both items have been used in previous research, and these events have been related to better daily mood and lower depressive symptoms in adolescents (Telzer & Fuligni, 2013). Separate sums were calculated for the number of days when participants reported getting along with their parents and when participants spent leisure time with their family, with 0 indicating that youth never reported each event and 15 indicating that they experienced that event every day.

### Analytic strategy

We used stepwise regression to determine whether frequency of family meals was related to substance use count and frequency of alcohol, marijuana, and cigarette use. To examine differences by gender, we also included Family Meal Frequency × Gender interactions as predictors. Significant interactions were probed in two ways: (1) testing the association between frequency of family meals and substance use at the levels of male adolescents and female adolescents and (2) testing how the effect of gender on substance use varied at different frequencies of family meals. With this second approach, we used the Johnson–Neyman technique to identify the regions of significance, or the values of family meal frequency at which gender differences in substance use emerged (Johnson & Fay, 1950).

We repeated analyses controlling for adolescent age, ethnicity, family income, and parents' education. Daily reports of getting along with parents and leisure time with family were included as additional control variables to determine whether family meal frequency, as opposed to time with family more generally, was uniquely related to substance use. In the final step, parental support and family cohesion were also included as predictors, and moderated mediation models with 20,000 bootstraps were used to determine whether associations between family meal frequency and substance use were explained by aspects of family relationships (Figure S1). Family meal frequency, age, family income, parents' education, frequency of getting along with parents, frequency of leisure time spent with family, parental support, and family cohesion were mean‐centered. Gender was dummy‐coded (male = 0, female = 1), and ethnicity was dummy‐coded with Latine (sample majority) as the reference group. Analyses were tested in Stata 16.1. All models used full‐information maximum likelihood to estimate missing data.^1^

## RESULTS

### Descriptive statistics

Rates of substance use were low in this sample (Table 1). Substance use count and frequency did not differ between male and female participants, *ps* > 0.08. Older adolescents reported higher substance use count and more frequent use of each substance (Table 2).

**TABLE 1:** Frequencies of lifetime substance use.

Variable	Percentage (%)
Substance use count	
0	48.39
1	24.84
2	15.48
3	6.45
4	3.87
5	0.97
Ever used alcohol	43.55
Ever used marijuana	23.55
Ever used cigarettes	14.19

**TABLE 2:** Descriptive Statistics and Correlations for Study Variables by Gender.

Variables	Male M(SD)	Female M(SD)	1.	2.	3.	4.	5.	6.	7.	8.	9.	10.	11.	12.
1. Family meal frequency	9.03 (4.47)	8.92 (4.54)	—	−0.33***	−0.25***	−0.26***	−0.32***	−0.04	0.10	0.07	0.22**	0.41***	0.41***	0.46***
2. Substance Use Count	1.05 (1.18)	0.88 (1.18)	0.03	—	0.68***	0.76***	0.72***	0.14	−0.06	−0.07	−0.18*	−0.25***	−0.08	−0.18*
3. Cigarette frequency	0.18 (0.70)	0.15 (0.38)	0.05	0.44***	—	0.35***	0.36***	0.09	−0.05	−0.07	0.00	−0.06	−0.09	−0.07
4. Alcohol frequency	1.51 (2.01)	1.48 (2.01)	−0.02	0.70***	0.27**	—	0.56***	0.13	−0.07	−0.15*	−0.20**	−0.28***	−0.03	−0.11
5. Marijuana frequency	1.08 (2.29)	0.69 (1.68)	0.03	0.69***	0.25**	0.57***	—	0.02	−0.05	−0.09	−0.09	−0.15	−0.03	−0.15*
6. Age	16.38 (0.76)	16.41 (0.73)	−0.07	0.27**	0.17	0.22*	0.28**	—	−0.11	−0.14	−0.04	0.00	0.06	−0.10
7. Income	75.77 (82.98)	65.39 (71.38)	0.01	−0.15	−0.08	−0.02	−0.02	0.06	—	0.34***	0.04	0.05	0.12	0.02
8. Parents' education	7.48 (1.73)	6.99 (1.84)	0.02	−0.12	−0.17*	0.02	0.04	−0.09	0.30***	—	0.00	0.04	0.13	0.14
9. Parental support	3.68 (0.98)	3.64 (0.94)	0.31***	−0.05	0.06	−0.09	0.13	−0.03	0.14	0.07	—	0.70***	0.36***	0.22**
10. Family cohesion	3.72 (0.70)	3.70 (0.77)	0.36***	−0.02	0.08	−0.09	0.04	−0.05	0.15	0.07	0.76***	—	0.40***	0.24**
11. Family leisure frequency	6.32 (4.62)	6.52 (5.04)	0.50***	0.01	0.12	−0.02	−0.01	0.06	0.06	0.12	0.30***	0.34***	—	0.34***
12. Get along with parents frequency	10.80 (4.07)	12.01 (3.48)	0.32***	0.02	−0.01	−0.01	0.00	−0.08	0.13	0.05	0.37***	0.35***	0.35***	—

*Note*: *n* = 132 for males, *n* = 178 for females. Correlations for male adolescents are below the diagonal, and correlations for female adolescents are above the diagonal. A significant difference in means only emerged for frequency of getting along with parents, *t*(308) = 2.81, *p* = 0.005.

*p < 0.05, **p < 0.01, ***p < 0.001.

Almost all adolescents reported having a family meal at least once during the study period, and participants reported family meals on slightly over half of the days (Table 2). Family meal frequency did not differ by gender, ethnicity, age, family income, or parents' education, *ps* > 0.18. Participants reported high levels of parental support and family cohesion, and reported getting along with their parents on the majority of days and spending leisure time with family on slightly under half of the days. Correlations indicated that adolescents who had more family meals reported moderately higher parental support, higher family cohesion, more frequently getting along with their parents, and more frequently spending leisure time with their family on average (Table 2). Among female adolescents, higher parental support and family cohesion were weakly associated with lower substance use count and less frequent alcohol use, and higher frequency of getting along with parents was weakly associated with lower substance use count and less frequent marijuana use. No significant associations emerged for male adolescents.

### Family meal frequency and substance use

**FIGURE 1:**
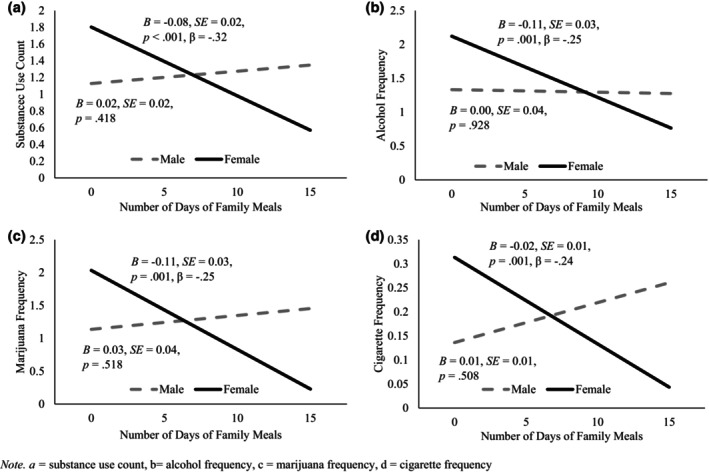
Substance use count (a), alcohol frequency (b), marijuana frequency (c), and cigarette frequency (d) as a function of family meals and gender.

Regression models tested whether frequency of family meals was related to substance use, and whether associations differed by gender. Results indicated gender differences in the associations between family meal frequency and substance use count (*B* = −0.09, SE = 0.03, 95% Confidence Interval [CI] [−0.15, −0.04], *p* = 0.001, *β* = −0.23), frequency of alcohol use (*B* = −0.11, SE = 0.05, 95% CI [−0.20, −0.01], *p* = 0.031, *β* = −0.16), frequency of marijuana use (*B* = −0.13, SE = 0.05, 95% CI [−0.23, −0.04], *p* = 0.006, *β* = −0.20), and frequency of cigarette use (*B* = −0.03, SE = 0.01, 95% CI [−0.06, −0.002], *p* = 0.033, *β* = −0.16). When interactions were probed at the level of gender, associations were consistently significant in female adolescents and remained significant after adjusting for participant age, ethnicity, income, and parents' education (Figure 1; Table 3, Table S1). In turn, associations between family meal frequency and substance use outcomes among male adolescents were consistently null in both unadjusted and adjusted analyses, *ps >* 0.46.

**TABLE 3:** Substance use count, alcohol frequency over the past year, marijuana frequency over the past year, and cigarette frequency over the past month as a function of family meal frequency and gender.

	Substance use count		Frequency of alcohol use		Frequency of marijuana use		Frequency of cigarette use	
	B	SE	B	SE	B	SE	B	SE
Intercept	0.80***	0.14	1.49***	0.24	0.75**	0.23	0.15*	0.06
Family meal frequency	−0.08***	0.02	0.11*	0.05	0.13**	0.05	0.03*	0.01
Female	0.19	0.13	−0.11***	0.03	−0.11***	0.03	−0.02*	0.01
Family meal frequency × female	0.09**	0.03	0.02	0.22	0.37	0.22	0.04	0.06
Income	−0.01	0.01	−0.01	0.02	−0.01	0.02	0.00	0.00
Age	0.32***	0.09	0.45	0.15	0.42**	0.15	0.08*	0.04
Parents' education	−0.01	0.04	−0.03	0.07	0.01	0.07	−0.03	0.02
Asian	−0.15	0.19	−0.52	0.33	−0.55	0.33	−0.11	0.09
European American	0.27	0.17	0.27	0.28	0.22	0.28	0.02	0.08
Different identity	−0.10	0.29	−0.48	0.49	−0.33	0.49	0.29*	0.13

*Note*. Family Meal Frequency, Income, Age, and Parents' Education were centered at the sample mean. Female was dummy‐coded (0 = male, 1 = female). Ethnicity was dummy‐coded with Latine as the reference group.

*p < 0.05, **p < 0.01, ***p < 0.001.

Next, we used the Johnson–Neyman technique to determine how gender differences in substance use varied by frequency of family meals. This technique involves identifying the region of significance, or the number of family meals at which gender differences in substance use emerge. Regarding substance use count, female adolescents used more substances than male adolescents among youth who had family meals on between 0 and 2 of the 15 possible days (*B* = −0.68, SE = 0.30, *p* = 0.023 for 0 days; *B* = −0.48, SE = 0.25, *p* = 0.050 for 2 days). However, female adolescents used fewer substances than male adolescents among youth who had family meals on 10 or more days (*B* = 0.29, SE = 0.13, *p* = 0.030 for 10 days; *B* = 0.78, SE = 0.22, *p* < 0.001 for 15 days). Female adolescents also used marijuana less frequently than male adolescents among youth who had family meals on 10 or more days (*B* = 0.52, SE = 0.23, *p* = 0.025 for 10 days; *B* = 1.23, SE = 0.37, *p* = 0.001 for 15 days) and used cigarettes less frequently than male adolescents among youth who had family meals on 13 or more days (*B* = 0.16, SE = 0.08, *p* = 0.046 for 13 days; *B* = 0.22, SE = 0.10, *p* = 0.035 for 15 days). Lastly, alcohol use did not differ significantly between male and female adolescents at any level of family meal frequency, *ps >* 0.07.

### Uniqueness of family meal frequency and substance use associations

To determine whether family meals were unique from other daily experiences, associations between family meal frequency and substance use were tested controlling for the number of days when adolescents reported getting along with parents and spending leisure time with their family, as indicated by daily checklists. The Family Meal Frequency × Gender interactions remained significant for all outcomes (*p*s between 0.001 and 0.042, *β*s between 0.15 and 0.23), and neither of the other daily family experiences were related to substance use across the sample or in analyses stratified by gender (Tables S2, S3).

Parental support and family cohesion were additionally tested as predictors of substance use in the full sample and in female adolescents to identify potential mechanisms relating family meal frequency to substance use.^2^ The Family Meal Frequency × Gender interactions remained significant for all outcomes (Table S4). Simple slopes in female adolescents indicated that associations between more frequent family meals and lower substance use outcomes among female adolescents remained significant over and above parental support and family cohesion (*p*s between 0.001 and 0.015, *β*s between −0.20 and 0.35; Table S5). Higher parental support was related to lower substance use count (*B* = −0.19, SE = 0.09, 95% CI [−0.38, −0.01], *p* = 0.039, *β* = −0.15) and frequency of alcohol use among girls (*B* = −0.46, SE = 0.16, 95% CI [−0.77, −0.14], *p* = 0.039, *β* = −0.15), over and above family meal frequency. Higher family cohesion was similarly associated with lower substance use count (*B* = −0.30, SE = 0.12, 95% CI [−0.53, −0.06], *p* = 0.014, *β* = −0.19) and less frequent alcohol use (*B* = −0.46, SE = 0.16, 95% CI [−1.12, −0.32], *p* = 0.004, *β* = −0.21). Associations emerged for neither marijuana nor cigarette frequency (all *p*s >0.25). We therefore used conditional process analysis to determine whether family meal frequency was related to substance use count and frequency of alcohol use through overlapping pathways (i.e., shared variance) with parental support and family cohesion, and the degree to which these pathways differed by gender (Figure S1a). Models did not indicate a significant gender difference in the magnitude of the indirect association. Therefore, there was no evidence that parental support or family cohesion statistically accounted for gender differences in the association between family meal frequency and substance use outcomes. In models that did not specify moderation of the indirect effect (Figure S1b), significant indirect associations emerged suggesting that higher family meal frequency and family cohesion were related to lower substance use count (*ab* = −0.01, SE = 0.005, 95% CI [−0.02, −0.0001]; 20.2% of the total association for family meal frequency among female adolescents) and less frequent alcohol use (*ab* = −0.02, SE = 0.01, *p* = 0.038, 95% CI [−0.04, −0.009]; 31.1% of the total association) through overlapping pathways. In addition to these shared indirect associations, frequency of family meals showed an independent, direct association with both substance use measures that significantly differed by gender. No other indirect associations were observed across models (Table S6).

## DISCUSSION

Although youth who have more frequent family meals tend to show better well‐being (Utter et al., 2017), associations have been less consistent for adolescent substance use (Goldfarb et al., 2015). Limited research has assessed whether these associations differ by gender and are explained by family relationships and other daily experiences among high school students. As hypothesized, female adolescents who had more frequent family meals reported lower substance use count and less frequent use of substances, but associations were not found for male adolescents. Interestingly, other daily events (i.e., getting along with parents, leisure time with family) were unrelated to substance use. Family meal frequency and family cohesion were related to substance use count and frequency of alcohol use through a shared pathway, although parental support and family cohesion did not appear to explain gender‐specific associations between family meal frequency and substance use measures. Rather, family meal frequency consistently showed a unique direct association with substance use in female adolescents that was independent of parental support and family cohesion. Taken together, our results suggest that family meals may uniquely relate to lower substance use for female adolescents.

In line with prior studies, family meals were related to lower substance use only among female adolescents (Eisenberg et al., 2008; Fisher et al., 2007; White & Halliwell, 2011). Substance use tends to increase during high school, and the present study suggested that more frequent family meals may be related to lower substance use risk during this time, replicating results from a previous study of high school students (Eisenberg et al., 2004). Because we lacked earlier assessments of family meals and substance use, it is possible that observed results were driven by youth who had frequent family meals consistently throughout their childhood. Future interventions can assess whether initiation of daily family meals can reduce substance use risk and frequency among female adolescents, irrespective of family meal frequency in childhood.

Results also suggested that family meals were uniquely related to lower substance use for female adolescents, controlling for daily instances of getting along with parents and family leisure. Female youth who rarely had family meals used more substances than male youth, whereas female youth who frequently had family meals used fewer substances than male youth. It is possible that family meals are distinct from other daily family events because they can serve as a regular family ritual that involves discussion and thereby enable parents to monitor their children (Fiese et al., 2002; Fiese et al., 2006; Skeer et al., 2018). Youth may avoid using substances if they anticipate interacting with their parents, in line with evidence that family meals are related to less frequent alcohol use through parental involvement (Perasso et al., 2021).

Parental support and family cohesion were both related to substance use count and frequency of alcohol use, but not frequency of marijuana or cigarette use. These substance‐specific findings may have emerged statistically because adolescents in this sample were more likely to use alcohol than marijuana or cigarettes. Indirect associations emerged such that family meal frequency and family cohesion were related to lower substance use count and alcohol use through overlapping pathways. The present study was cross‐sectional, precluding assessment of temporal pathways. Theoretically, adolescents who have family meals more frequently may have higher family cohesion as previously posited (Goldfarb et al., 2015; White & Halliwell, 2010), but it is also possible that families with higher cohesion tend to dine together, thereby reducing the risk of substance use. However, family cohesion did not account for the majority of the association between family meal frequency and substance use in female adolescents, and having more frequent family meals surprisingly remained associated with lower levels of all substance use outcomes in female adolescents after accounting for other family‐related variables.

Taken together, gender‐specific associations between family meal frequency and substance use were not generally explained by parental support or family cohesion, at odds with previous evidence that family‐related variables including cohesion explain the relationship between family meals and substance use (Goldfarb et al., 2015; White & Halliwell, 2010). Differences may have emerged because the present study incorporated a daily checklist protocol. Adolescents reported whether they experienced a family meal and other family events (i.e., parental support, family leisure) daily, which may have improved accuracy of estimates of family meals compared to other methodologies that may be more susceptible to recall bias such as retrospective reporting. This methodology also allowed us to identify whether adolescents' substance use was differentially related to family meals versus family time more generally. These findings suggest that interventions targeted at reducing substance use in female adolescents may benefit from reducing barriers to family dinners (e.g., scheduling conflicts; lack of time, energy, or resources) and building routines around family meals (e.g., Family Dinner Project, Family Day) as opposed to incorporating other family activities (Middleton et al., 2020; Utter et al., 2019). Given that family meals were related to lower substance use independently from parental support and cohesion, interventions could incorporate multiple arms that independently promote family meals versus family support and cohesion.

Family meals may relate to substance use among female but not male adolescents because of differences in substance use motives and sensitivity to family events. Consistent family meals may signal parents' investment in their adolescents, so that adolescents can comfortably discuss interpersonal problems and changes in their friend groups that may precede substance use (Crouter et al., 2004; Dishion & Owen, 2009; Koblinsky et al., 2006). Such discussions may promote emotion regulation and thereby reduce stress (Armstrong‐Carter & Telzer, 2020; Hansson et al., 2017; Romano et al., 2022). Meals may thereby reduce female adolescents' inclination to use substances, as female adolescents are more motivated to use substances to relieve stress, whereas male adolescents are more motivated to use substances for social and emotional enhancing effects (Kuntsche et al., 2015; Pompili & Laghi, 2019). Female adolescents also tend to be more relational and sensitive to positive and negative family dynamics than male adolescents (Crosnoe, 2012; Keijsers et al., 2010). Indeed, we found that female adolescents got along with their parents more often than male adolescents, and higher parental support, family cohesion, and frequency of getting along with parents were correlated with lower substance use count and frequency among female but not male adolescents. Gender‐specific associations have emerged because female adolescents may benefit more from disclosure and communication that is involved in family relationships (Keijsers et al., 2010). Alternatively, it is possible that youth who have more family meals may worry about punishment from their parents or have greater time restrictions related to evening activities that limit possible engagement with substance use (Perasso et al., 2021), although this pathway seems unlikely given observed gender differences in associations and given that other daily family experiences did not similarly explain associations.

Family meals may not be related to substance use among male adolescents because they may be less inclined to utilize time during family dinners to describe their days, they may appraise these meals more negatively than female adolescents, and they may use substances for reasons that cannot be addressed by family meals. Male adolescents tend to be lower in emotional self‐disclosure than female adolescents (Papini et al., 1990). As a result, they may not express themselves at family dinners or value time spent with family to the same degree as female adolescents. Aspects of family meals can be modified so that male adolescents can be more actively involved in preparing the meal (e.g., cooking, setting up, cleaning afterward), in line with evidence that engagement in family assistance is related to lower substance use in adolescents (Telzer et al., 2014). Because conversations at family meals often center around food (Ochs et al., 1996), interventions can be developed to not only support having family meals as a consistent ritual but also help parents, especially fathers, facilitate discussions specifically regarding their children's lives and social activities.

Additionally, male adolescents are often more inclined to use substances for social benefits (Kuntsche et al., 2015), which may not be addressed by family meals. It is possible that adolescent boys are more susceptible to deviant peer pressure than girls (McCoy et al., 2019), potentially related to prescriptive gender stereotypes and their endorsement of traditionally masculine norms such as risk‐taking (Iwamoto & Smiler, 2013). Because male adolescents are at heightened risk for exposure to substance use from peers than female adolescents (Johnston et al., 2023), family meal frequency earlier in development (e.g., middle school) may be more important for male adolescents' substance use than current family meal frequency (Eisenberg et al., 2004, 2008). Future research should investigate how social factors (e.g., endorsement of social norms, motivation for peer status) and academic factors (e.g., academic value, grades) can influence male adolescents' substance use, and whether familial involvement can reduce male adolescents' substance use by intervening upon those factors.

### Limitations

Study findings should be interpreted in the context of limitations. We could not assess directionality of associations, and there was discordance in the timescale of measurement with participants reporting prior substance use and frequency of family meals over 2 weeks. Although family meals have been prospectively related to substance use (Eisenberg et al., 2008), it is possible that adolescents' substance use could result in consequences that interfere with their daily family rituals, similar to how having parents with substance use disorders can affect family relationships (Chassin et al., 2019). Youth in this sample had low rates of substance use and generally reported using for a few days in a given year, which may not be severe enough to disrupt daily family rituals or undermine family relationships, although prospective assessments will be needed to test this possibility. Substance use was also not assessed daily, precluding assessment of whether family meals were related to substance use at the daily level. Daily family meals can differ with respect to time of day, location, and number of family members present. These factors may influence the degree to which family meals relate to substance use. Similarly, additional information regarding the context of adolescents' substance use, such as substance use motivation (e.g., promote positive arousal, reduce distress) and whether adolescents use alone or with others, including friends and siblings, may clarify gender differences in observed associations. The present study used lifetime count of substance use and self‐reported frequency of use, which may have resulted in biased recall of substance use frequency compared to daily reports. Participants were asked to report use of alcohol and marijuana over the past year such that assessments could account for potential differences in rates of substance use across the academic year (i.e., differences in use by season, school year vs summer). However, we also measured cigarette use over the past month, and these reports may be less representative of adolescents' overall use and potentially more influenced by time of year compared to the other substance use outcomes. Differences in associations between family meal frequency and substance use are likely to be due to differences in socialization rather than biology. The measure of gender was flawed because the response options corresponded to biological sex. Gender and sex often do not correspond, and future studies should assess gender with open‐ended responses (Westbrook & Saperstein, 2015). Finally, the landscape of adolescent substance use has changed since 2011–2012 when these data were collected. Prevalence of alcohol and cigarette use in high school students has declined, particularly with the onset of the COVID‐19 pandemic, and other forms of use such as vaping have become more prevalent (Johnston et al., 2023). Therefore, findings should be replicated in more recently collected data.

## CONCLUSIONS

In conclusion, frequent family meals were related to lower substance use in female but not male adolescents, distinctly from other aspects of family relationships and daily activities. Family meals may be a stable family ritual when parents can be involved and discuss daily experiences with their youth. Our findings provide preliminary evidence that parents may be able to reduce female adolescents' substance use risk with respect to substance use count and frequency of alcohol, marijuana, and cigarette use by having more frequent family meals during high school. Other daily family events, parental support, and family cohesion did not account for associations between family meal frequency and substance use in female adolescents, suggesting that interventions should consider promoting family meal frequency in addition to other family experiences. Future research should identify aspects of family meals that directly reduce female adolescents' substance use, the daily processes by which family meals relate to substance use, and factors that can reduce male adolescents' substance use (e.g., peer or school factors).

## FUNDING INFORMATION

This research was supported by NIH National Center for Advancing Translational Science (NCATS) UCLA CTSI (UL1TR001881) and funding from the Eunice Kennedy Shriver National Institute of Child Health and Human Development (R01‐HD062547), the UCLA California Center for Population Research (P2C‐HD041022), the UCLA Older Americans Independence Center (P30‐AG028748), and the USC/UCLA Center for Biodemography and Population Health (P30‐AG017265). Danny Rahal was supported by the National Institute on Drug Abuse of the National Institutes of Health under Award Number F31DA051181 and with support from the Prevention and Methodology Training program (T32 DA017629; MPIs S. Lanza and J. Maggs). The content is solely the responsibility of the authors and does not necessarily represent the official views of the National Institutes of Health.

The authors would like to thank the participants for their involvement in this study, as well as Narissa Carthy‐Dundas, Zahara Cuevas‐Kovanis, and Violet Kwan for their efforts in proofreading the manuscript.

## Supporting information


Appendix S1.

Appendix S2.

## Footnotes

^1^
Six participants did not provide substance use data. These six participants did not differ from the analytic sample by age, gender, ethnicity, family income, or parents' education, *ps >* 0.23. An identical pattern of results emerged without full‐information maximum likelihood (*n* = 310).

^2^
Parental support and family cohesion were tested in separate models due to their high inter‐correlation (*r*[314] = 0.73, *p* < 0.001). Associations between family meal frequency and substance use outcomes were unaffected by testing variables simultaneously versus separately.
