# Supplementary material for: Family meals are associated with lower substance use in female adolescents
Source: Fam Process. 2024 Jul 31;64(1):e13039. doi: 10.1111/famp.13039 (PMC11781996; doi:10.1111/famp.13039)
Supplement: Supplementary file 1 — Appendix S1. [file FAMP-64-0-s002.docx]

**Supplemental Material A**

Supplemental Table A1 presents associations between family meal frequency and substance use in female adolescents over and above demographic variables. Supplemental Table A2 presents substance use as a function of family meal frequency and gender over and above demographic variables and daily family experiences. Supplemental Table A3 presents substance use as a function of family meal frequency over and above demographic variables and daily family experiences in female adolescents. Supplemental Table A4 presents substance use as a function of family meal frequency and gender over and above demographic variables, daily family experiences, family cohesion, and parental support. Supplemental Table A5 presents substance use as a function of family meal frequency over and above demographic variables, daily family experiences, family cohesion, and parental support in female adolescents. Supplemental Table A6 present indirect associations and gender-specific direct associations between family meal frequency (statistical predictor), family cohesion and parental support (statistical mediator), and substance use (outcome).

**Table A1**

*Substance Use as a Function of Family Meal Frequency and Gender Over and Above Demographic Factors in Female Adolescents*

|  | Substance Use Count | | Frequency of Alcohol Use | | Frequency of Marijuana Use | | Frequency of Cigarette Use | |
| --- | --- | --- | --- | --- | --- | --- | --- | --- |
|  | *B* | *SE* | *B* | *SE* | *B* | *SE* | *B* | *SE* |
| Intercept | 0.89*** | 0.16 | 1.63*** | 0.28 | 0.98*** | 0.24 | 0.16** | 0.05 |
| Family Meal Frequency | -0.08*** | 0.02 | -0.11*** | 0.03 | -0.12*** | 0.03 | -0.02** | 0.01 |
| Income | 0.00 | 0.01 | -0.01 | 0.02 | -0.01 | 0.02 | 0.00 | 0.00 |
| Age | 0.23* | 0.11 | 0.29 | 0.20 | 0.00 | 0.16 | 0.05 | 0.04 |
| Parents' Education | -0.02 | 0.05 | -0.13 | 0.09 | -0.09 | 0.07 | -0.01 | 0.02 |
| Asian | -0.36 | 0.24 | -0.82 | 0.42 | -0.74* | 0.35 | -0.11 | 0.08 |
| European American | 0.15 | 0.22 | 0.07 | 0.38 | -0.29 | 0.32 | 0.04 | 0.07 |
| Different Identity | 0.08 | 0.38 | -0.38 | 0.63 | -0.20 | 0.55 | -0.07 | 0.12 |

*Note.* Family Meal Frequency, Income, Age, and Parents’ Education were centered at the sample mean. Ethnicity was dummy-coded with Latine as the reference group.

**p*<.05, ***p*<.01, ****p*<.001

**Table A2**

*Substance Use as a Function of Family Meal Frequency and Gender Over and Above Demographic Factors and Daily Family Experiences*

|  | Substance Use Count | | Frequency of Alcohol Use | | Frequency of Marijuana Use | | Frequency of Cigarette Use | |
| --- | --- | --- | --- | --- | --- | --- | --- | --- |
|  | *B* | *SE* | *B* | *SE* | *B* | *SE* | *B* | *SE* |
| Intercept | 0.78** | 0.27 | 1.35** | 0.47 | 0.78 | 0.46 | 0.15 | 0.13 |
| Family Meal Frequency | -0.08*** | 0.02 | 0.11* | 0.05 | 0.13** | 0.05 | 0.03* | 0.01 |
| Female | 0.19 | 0.13 | -0.11** | 0.03 | -0.12** | 0.03 | -0.02* | 0.01 |
| Family Meal Frequency × Female | 0.09** | 0.03 | 0.03 | 0.22 | 0.37 | 0.22 | 0.04 | 0.06 |
| Income | -0.01 | 0.01 | -0.01 | 0.02 | -0.01 | 0.02 | 0.00 | 0.00 |
| Age | 0.32*** | 0.09 | 0.44** | 0.15 | 0.41** | 0.15 | 0.08 | 0.04 |
| Parents' Education | -0.01 | 0.04 | -0.04 | 0.07 | 0.01 | 0.07 | -0.04 | 0.02 |
| Asian | -0.13 | 0.20 | -0.49 | 0.34 | -0.53 | 0.34 | -0.08 | 0.09 |
| European American | 0.28 | 0.17 | 0.29 | 0.29 | 0.22 | 0.28 | 0.03 | 0.08 |
| Different Identity | -0.10 | 0.29 | -0.47 | 0.49 | -0.32 | 0.49 | 0.30* | 0.13 |
| Frequency of Getting Along with Parents | 0.00 | 0.02 | 0.01 | 0.03 | 0.00 | 0.03 | 0.00 | 0.01 |
| Frequency of Family Leisure | 0.01 | 0.02 | 0.00 | 0.03 | 0.01 | 0.03 | 0.01 | 0.01 |

*Note.* Family Meal Frequency, Income, Age, Parents’ Education, Frequency of Getting Along with Parents, and Frequency of Family Leisure were centered at the sample mean. Female was dummy-coded (0 = male, 1 = female). Ethnicity was dummy-coded with Latine as the reference group.

**p*<.05, ***p*<.01, ****p*<.001

**Table A3**

*Substance Use as a Function of Family Meal Frequency and Gender Over and Above Demographic Factors and Daily Family Experiences in Female Adolescents*

|  | Substance Use Count | | Frequency of Alcohol Use | | Frequency of Marijuana Use | | Frequency of Cigarette Use | |
| --- | --- | --- | --- | --- | --- | --- | --- | --- |
|  | *B* | *SE* | *B* | *SE* | *B* | *SE* | *B* | *SE* |
| Intercept | 0.87** | 0.32 | 0.68* | 0.32 | 0.63* | 0.32 | 0.17 | 0.33 |
| Family Meal Frequency | -0.32*** | 0.08 | -0.28** | 0.08 | -0.35*** | 0.08 | -0.26** | 0.08 |
| Income | -0.02 | 0.08 | -0.03 | 0.08 | -0.03 | 0.08 | -0.02 | 0.08 |
| Age | 0.14 | 0.07 | 0.10 | 0.07 | -0.01 | 0.07 | 0.10 | 0.07 |
| Parents' Education | -0.02 | 0.08 | -0.13 | 0.08 | -0.11 | 0.08 | -0.03 | 0.08 |
| Asian | -0.12 | 0.09 | -0.16 | 0.10 | -0.16 | 0.09 | -0.13 | 0.10 |
| European American | 0.07 | 0.09 | 0.03 | 0.09 | -0.07 | 0.09 | 0.06 | 0.10 |
| Different Identity | 0.02 | 0.08 | -0.04 | 0.08 | -0.02 | 0.08 | -0.05 | 0.08 |
| Frequency of Getting Along with Parents | -0.04 | 0.08 | 0.03 | 0.08 | -0.02 | 0.08 | 0.08 | 0.08 |
| Frequency of Family Leisure | 0.04 | 0.08 | 0.05 | 0.08 | 0.11 | 0.08 | -0.03 | 0.09 |

*Note.* Family Meal Frequency, Income, Age, Parents’ Education, Frequency of Getting Along with Parents, and Frequency of Family Leisure were centered at the sample mean. Ethnicity was dummy-coded with Latine as the reference group.

**p*<.05, ***p*<.01, ****p*<.001

**Table A4**

*Substance Use as a Function of Family Meal Frequency and Gender Over and Above Demographic Factors, Daily Family Experiences, and Family Cohesion (above) and Parental Support (below)*

|  | Substance Use Count | | Frequency of Alcohol Use | | Frequency of Marijuana Use | | Frequency of Cigarette Use | |
| --- | --- | --- | --- | --- | --- | --- | --- | --- |
|  | *B* | *SE* | *B* | *SE* | *B* | *SE* | *B* | *SE* |
| Intercept | 0.72** | 0.27 | 1.22** | 0.46 | 0.76 | 0.47 | 0.15 | 0.13 |
| Family Meal Frequency | -0.07*** | 0.02 | 0.10* | 0.05 | 0.13** | 0.05 | 0.03* | 0.01 |
| Female | 0.19 | 0.13 | -0.09* | 0.03 | -0.11** | 0.03 | -0.02* | 0.01 |
| Family Meal Frequency × Female | 0.09** | 0.03 | 0.04 | 0.22 | 0.37 | 0.22 | 0.04 | 0.06 |
| Income | -0.01 | 0.01 | -0.01 | 0.02 | -0.01 | 0.02 | 0.00 | 0.00 |
| Age | 0.32*** | 0.09 | 0.44** | 0.15 | 0.41** | 0.15 | 0.08 | 0.04 |
| Parents' Education | -0.01 | 0.04 | -0.03 | 0.07 | 0.01 | 0.07 | -0.04 | 0.02 |
| Asian | -0.13 | 0.20 | -0.51 | 0.34 | -0.53 | 0.34 | -0.08 | 0.09 |
| European American | 0.31 | 0.17 | 0.37 | 0.28 | 0.24 | 0.29 | 0.03 | 0.08 |
| Different Identity | -0.08 | 0.29 | -0.44 | 0.48 | -0.32 | 0.49 | 0.30* | 0.13 |
| Frequency of Getting Along with Parents | 0.00 | 0.02 | 0.02 | 0.03 | 0.00 | 0.03 | 0.00 | 0.01 |
| Frequency of Family Leisure | 0.01 | 0.02 | 0.02 | 0.03 | 0.01 | 0.03 | 0.01 | 0.01 |
| Family Cohesion | -0.20* | 0.10 | -0.53** | 0.16 | -0.06 | 0.16 | 0.03 | 0.05 |
|  | *B* | *SE* | *B* | *SE* | *B* | *SE* | *B* | *SE* |
| Intercept | 0.72** | 0.27 | 1.22** | 0.46 | 0.82 | 0.47 | 0.15 | 0.13 |
| Family Meal Frequency | -0.08*** | 0.02 | 0.11* | 0.05 | 0.13** | 0.05 | 0.03* | 0.01 |
| Female | 0.20 | 0.13 | -0.11** | 0.03 | -0.12** | 0.03 | -0.02* | 0.01 |
| Family Meal Frequency × Female | 0.09** | 0.03 | 0.04 | 0.22 | 0.36 | 0.22 | 0.04 | 0.06 |
| Income | -0.01 | 0.01 | -0.01 | 0.02 | -0.01 | 0.02 | 0.00 | 0.00 |
| Age | 0.31*** | 0.09 | 0.43** | 0.15 | 0.42** | 0.15 | 0.08 | 0.04 |
| Parents' Education | -0.01 | 0.04 | -0.04 | 0.07 | 0.02 | 0.07 | -0.03 | 0.02 |
| Asian | -0.16 | 0.20 | -0.58 | 0.34 | -0.51 | 0.34 | -0.08 | 0.09 |
| European American | 0.28 | 0.17 | 0.30 | 0.28 | 0.22 | 0.28 | 0.03 | 0.08 |
| Different Identity | -0.08 | 0.29 | -0.43 | 0.48 | -0.33 | 0.49 | 0.30* | 0.13 |
| Frequency of Getting Along with Parents | 0.01 | 0.02 | 0.02 | 0.03 | -0.01 | 0.03 | 0.00 | 0.01 |
| Frequency of Family Leisure | 0.01 | 0.02 | 0.02 | 0.03 | 0.01 | 0.03 | 0.01 | 0.01 |
| Parental Support | -0.14* | 0.07 | -0.32** | 0.12 | 0.08 | 0.12 | 0.01 | 0.03 |

*Note.* Family Meal Frequency, Income, Age, Parents’ Education, Frequency of Getting Along with Parents, Frequency of Family Leisure, Family Cohesion, and Parental Support were centered at the sample mean. Female was dummy-coded (0 = male, 1 = female). Ethnicity was dummy-coded with Latine as the reference group.

**p*<.05, ***p*<.01, ****p*<.001

**Table A5**

*Substance Use as a Function of Family Meal Frequency and Gender Over and Above Demographic Factors, Daily Family Experiences, and Family Cohesion (above) and Parental Support (below) in Female Adolescents*

|  | Substance Use Count | | Frequency of Alcohol Use | | Frequency of Marijuana Use | | Frequency of Cigarette Use | |
| --- | --- | --- | --- | --- | --- | --- | --- | --- |
|  | *B* | *SE* | *B* | *SE* | *B* | *SE* | *B* | *SE* |
| Intercept | 1.01** | 0.37 | 1.31* | 0.63 | 1.04 | 0.54 | 0.07 | 0.12 |
| Family Meal Frequency | -0.08*** | 0.02 | -0.12** | 0.04 | -0.13*** | 0.03 | -0.02** | 0.01 |
| Income | 0.00 | 0.01 | -0.01 | 0.02 | -0.01 | 0.02 | 0.00 | 0.00 |
| Age | 0.21 | 0.11 | 0.26 | 0.19 | -0.04 | 0.16 | 0.05 | 0.04 |
| Parents' Education | -0.02 | 0.05 | -0.16 | 0.08 | -0.10 | 0.07 | -0.01 | 0.02 |
| Asian | -0.39 | 0.25 | -0.88* | 0.43 | -0.65 | 0.37 | -0.11 | 0.08 |
| European American | 0.13 | 0.22 | 0.06 | 0.38 | -0.25 | 0.32 | 0.04 | 0.07 |
| Different Identity | 0.11 | 0.38 | -0.31 | 0.62 | -0.11 | 0.55 | -0.07 | 0.13 |
| Frequency of Getting Along with Parents | -0.01 | 0.03 | 0.03 | 0.05 | -0.01 | 0.04 | 0.01 | 0.01 |
| Frequency of Family Leisure | 0.02 | 0.02 | 0.05 | 0.04 | 0.05 | 0.03 | 0.00 | 0.01 |
| Family Cohesion | -0.19* | 0.09 | -0.46** | 0.16 | -0.15 | 0.13 | 0.02 | 0.03 |
|  | *B* | *SE* | *B* | *SE* | *B* | *SE* | *B* | *SE* |
| Intercept | 1.02** | 0.37 | 1.35* | 0.62 | 1.06* | 0.54 | 0.07 | 0.12 |
| Family Meal Frequency | -0.07** | 0.02 | -0.09* | 0.04 | -0.12*** | 0.03 | -0.02** | 0.01 |
| Income | 0.00 | 0.01 | -0.01 | 0.02 | -0.01 | 0.02 | 0.00 | 0.00 |
| Age | 0.22* | 0.11 | 0.29 | 0.19 | -0.03 | 0.16 | 0.05 | 0.04 |
| Parents' Education | -0.02 | 0.05 | -0.14 | 0.08 | -0.10 | 0.07 | -0.01 | 0.02 |
| Asian | -0.36 | 0.25 | -0.80 | 0.43 | -0.62 | 0.37 | -0.11 | 0.08 |
| European American | 0.18 | 0.22 | 0.17 | 0.37 | -0.21 | 0.32 | 0.04 | 0.07 |
| Different Identity | 0.11 | 0.38 | -0.31 | 0.62 | -0.11 | 0.55 | -0.07 | 0.13 |
| Frequency of Getting Along with Parents | -0.01 | 0.03 | 0.02 | 0.05 | -0.01 | 0.04 | 0.01 | 0.01 |
| Frequency of Family Leisure | 0.02 | 0.02 | 0.05 | 0.04 | 0.05 | 0.03 | 0.00 | 0.01 |
| Parental Support | -0.30* | 0.12 | -0.72*** | 0.20 | -0.15 | 0.17 | 0.01 | 0.04 |

*Note.* Family Meal Frequency, Income, Age, Parents’ Education, Frequency of Getting Along with Parents, Frequency of Family Leisure, Family Cohesion, and Parental Support were centered at the sample mean. Ethnicity was dummy-coded with Latine as the reference group.

**p*<.05, ***p*<.01, ****p*<.001

**Table A6**

*Mediation Models Testing Indirect Associations Between Family Meal Frequency and Substance Use Measures Through Shared Variance with Family Cohesion (top) and Parental Support (bottom)*

| **Outcomes** | **Indirect Association** | **Direct Association in Female Adolescents** |
| --- | --- | --- |
| **Family Cohesion** |  |  |
| Substance Use Count* | *ab* = -0.009, *SE* = 0.005, 95% CI [-0.02, -0.0001] | *ab* = -0.08, *SE* = 0.02, 95% CI [-0.12, -0.03] |
| Alcohol* | *ab* = -0.02, *SE* = 0.009, 95% CI [-0.04, -0.01] | *ab* = -0.09, *SE* = 0.04, 95% CI [-0.16, -0.02] |
| Marijuana | *ab* = -0.002, *SE* = 0.007, 95% CI [-0.02, 0.01] | *ab* = -0.12, *SE* = 0.04, 95% CI [-0.19, -0.05] |
| Cigarettes | *ab* = 0.001, *SE* = 0.002, 95% CI [-0.003, 0.005] | *ab* = -0.02, *SE* = 0.01, 95% CI [-0.04, -0.002] |
| **Parental Support** |  |  |
| Substance Use Count | *ab* = -0.003, *SE* = 0.003, 95% CI [-0.009, 0.001] | *ab* = -0.08, *SE* = 0.02, 95% CI [-0.13, -0.04] |
| Alcohol | *ab* = -0.008, *SE* = 0.006, 95% CI [-0.02, 0.001] | *ab* = -0.11, *SE* = 0.04, 95% CI [-0.18, -0.04] |
| Marijuana | *ab* = 0.002, *SE* = 0.004, 95% CI [-0.005, 0.01] | *ab* = -0.12, *SE* = 0.04, 95% CI [-0.19, -0.05] |
| Cigarettes | *ab* = 0.0002, *SE* = 0.0009, 95% CI [-0.001, 0.002] | *ab* = -0.02, *SE* = 0.01, 95% CI [-0.04, -0.002] |

*Note.* *Indirect associations were significant based on 95% confidence intervals with 20000 bootstraps. All direct associations between family meal frequency and substance use measures in female adolescents were significant at α = .05. All direct associations between family meal frequency and substance use measures in male adolescents were not significant. Models controlled for Income, Age, Parents’ Education, Frequency of Getting Along with Parents, Frequency of Family Leisure. Ethnicity was dummy-coded with Latine as the reference group.

**Figure A1**

*Conceptual Mediation Models Testing Indirect Associations Between Family Meal Frequency, Family Cohesion and Parental Support, and Substance Use Measures*

 a)

Sex

Frequency of Family Meals

Family Cohesion/
Parental Support

b)

Sex

Substance Use Measures

Frequency of Family Meals

Family Cohesion/
Parental Support

Substance Use Measures

*Note*. Models tested moderation of the direct association between frequency of family meals and substance use measures and of the indirect association by gender (a), and tested moderation of the direct association between frequency of family meals and substance use measures by gender, but not the indirect association (b). Family cohesion and parental support were tested in separate models. Gender was dummy-coded (male = 0, female = 1). Models were tested with 20,000 bootstraps. Models were cross-sectional and therefore used to test overlapping pathway rather than truly indirect effects.
