# Supplementary material for: Family meals are associated with lower substance use in female adolescents
Source: Fam Process. 2024 Jul 31;64(1):e13039. doi: 10.1111/famp.13039 (PMC11781996; doi:10.1111/famp.13039)
Supplement: Supplementary file 2 — Appendix S2. [file FAMP-64-0-s001.docx]

# Supplemental Information B

# Parents’ Education

One caregiver reported for each parent and values were averaged across parents.

How far have you ever gone in school?

some elementary school  trade or vocational school

completed elementary school  some college

some junior-high school  graduated from college

completed junior-high school  some medical, law, or graduate school

some high school  graduated from medical, law, or graduate school

graduated from high school

# Daily Measures

Each day was assigned 1 (yes) or 0 (no) and a sum was calculated across days.

**Did any of the following activities or events occur to you today? (check if YES)**

O ate a meal with your family

O spent leisure time with your family

O got along with your parents

# Parental Support

**Please tell us how often each of these statements is true for you, that is, how often each statement describes you or your thoughts/feelings about your parents in the past month.**

*Use these numbers to answer the questions on this page:*

1 2 3 4 5

almost never once in a while sometimes frequently almost always

1. My parents respected my feelings.

2. My parents helped me understand myself better.

3. I told my parents about my worries and problems.

4. My parents helped me talk about my problems and difficulties.

5. My parents showed that (he/she) understands me.

6. When I was angry about something, my parents tried to be understanding.

7. I trusted my parents.

8. I could count on my parents when I needed to talk

9. If my parents knew something was bothering me, he/she asked me about it.

# Family Cohesion

**These questions ask you to think about how often you feel a certain way or do certain things with your family. Please circle the appropriate number.**

*Use these numbers to answer the questions on this page:*

1 2 3 4 5

almost never once in a while sometimes frequently almost always

1. My family and I are supportive of each other during difficult times. ------ 1 2 3 4 5

2. My family and I feel very close to each other. -----------------------------------1 2 3 4 5

3. It's easier to discuss problems with other people than with my family.---- 1 2 3 4 5

4. I feel closer to other people than to my family.-----------------------------------1 2 3 4 5

5. My family and I do things together.----------------------------------------------- 1 2 3 4 5

6. My family and I like to spend our free time with each other. --------------- 1 2 3 4 5

7. My family and I gather together in the same room. ---------------------------- 1 2 3 4 5

8. My family and I avoid each other at home. -------------------------------------- 1 2 3 4 5

9. My family and I have difficulty thinking of things to do together. ---------- 1 2 3 4 5

10. My family and I share interests and hobbies with each other.--------------1 2 3 4 5

**Substance Use Count**

Participants who reported any option besides that they had never used the substance were scored (1) for that substance, and a sum was calculated across the substances (possible range 0 to 7).

If you have ever smoked more than one or two puffs of a cigarette, how old were you when you smoked that much for the first time?

I have never smoked more than one or two puffs of a cigarette

Less than 9 years old

9 or 10 years old

11 or 12 years old

13 or 14 years old

15 or 16 years old

17 years old or older

If you have ever had more than a few sips of alcohol, how old were you when you first drank that much alcohol?

I have never had a drink of alcohol other than a few sips

Less than 9 years old

9 or 10 years old

11 or 12 years old

13 or 14 years old

15 or 16 years old

17 years old or older

If you have ever tried marijuana (pot, weed, grass, hash, etc.), how old were you when you tried it for the first time?

I have never tried marijuana

Less than 9 years old

9 or 10 years old

11 or 12 years old

13 or 14 years old

15 or 16 years old

17 years old or older

If you have ever tried any form of cocaine, including powder, crack, or freebase, how old were you when you tried it for the first time?

I have never tried cocaine

Less than 9 years old

9 or 10 years old

11 or 12 years old

13 or 14 years old

15 or 16 years old

17 years old or older

If you have ever used crystal meth (also called "ice" or “glass”), how old were you when you used crystal meth for the first time?

I have never tried crystal meth

Less than 9 years old

9 or 10 years old

11 or 12 years old

13 or 14 years old

15 or 16 years old

17 years old or older

If you have ever used any other type of illegal drug, such as LSD, PCP, ecstasy, mushrooms, speed, or heroin, how old were you when you used them for the first time?

I have never tried any other illegal drugs

Less than 9 years old

9 or 10 years old

11 or 12 years old

13 or 14 years old

15 or 16 years old

17 years old or older

If you have ever used any prescription drug such Ritalin, oxycotin, adderall, a valium, any narcotic, or any tranquilizer without a prescription, how old were you when you used a prescription drug without a prescription for the first time?

I have never used prescription drugs

Less than 9 years old

9 or 10 years old

11 or 12 years old

13 or 14 years old

15 or 16 years old

17 years old or older

**Substance Use Frequency**

Frequency of use of each item was assessed with a single item. Alcohol and marijuana were assessed over the past year, and cigarette use was assessed over the past 30 days. Unfortunately cigarette use was not assessed over the past year, resulting in a difference in the examined timescales.

During the past year, on how many days did you have at least one drink of alcohol?

1 or 2 days in the past year

3 to 11 days in the past year

one day a month

2 to 3 days a month

one day a week

two days a week

3 to 4 days a week

5 to 6 days a week

Every day

During the past year, how often did you use marijuana?

0 days in the past year

1 or 2 days in the past year

3 to 11 days in the past year

1 day a month

2 to 3 days a month

1 day a week

2 days a week

3 to 4 days a week

5 to 6 days a week

Every day

During the past 30 days, how many days did you smoke cigarettes?

0 days

1 or 2 days

3 to 5 days

6 to 9 days

10 to 19 days

20 to 29 days

0 All 30 days
